# Effects of Butylphthalide Combined with Fasudil on Inflammatory Factors, Cognitive Function and Vascular Endothelial Function in Patients with Subarachnoid Hemorrhage Complicated with Cerebral Vasospasm

**DOI:** 10.12669/pjms.39.6.6768

**Published:** 2023

**Authors:** Nan Tian, Ning Gan, Chaoyan Song, Fei Su, Jing Xie, Yu Zhang

**Affiliations:** 1 Nan Tian, Department of Neurosurgery, Baoding No.1 Central Hospital, Baoding 071000, Hebei, China; 2 Ning Gan, Department of Neurosurgery, Baoding No.1 Central Hospital, Baoding 071000, Hebei, China; 3 Chaoyan Song, Department of Neurosurgery, Baoding No.1 Central Hospital, Baoding 071000, Hebei, China; 4 Fei Su, Department of Neurosurgery, Baoding No.1 Central Hospital, Baoding 071000, Hebei, China; 5 Jing Xie, Department of Neurosurgery, Baoding No.1 Central Hospital, Baoding 071000, Hebei, China; 6 Yu Zhang, Department of Neurosurgery, Baoding No.1 Central Hospital, Baoding 071000, Hebei, China

**Keywords:** Butylphthalide, Fasudil, Subarachnoid Hemorrhage, Cerebral Vasospasm, Vasodilation Function

## Abstract

**Objective:**

To investigate the effect of butylphthalide combined with fasudil in the treatment of subarachnoid hemorrhage (SAH) with cerebral vasospasm (CVS) on inflammatory factors, cognitive function and vascular endothelial function.

**Methods:**

It is a retrospective study in which a total of 104 patients with SAH with CVS admitted to Baoding First Central Hospital from July 2020 to February 2022 were selected and randomly divided into two groups by drawing lots. Patients in the control group were treated with basic symptomatic treatment, while those in the observation group were treated with butylphthalide soft capsule combined with fasudil hydrochloride injection on the basis of the control group. Before and after treatment, serum neuron specific enolase (NSE), tumor necrosis factor-α(TNF-α), interleukin-8(IL-8), hypersensitive C-reactive protein (CRP), Birmingham Cognitive Screen test (BCoS) score, serum soluble intercellular adhesion molecule-1(ICAM-1), serum endothelin-1(ET-1), vascular endothelial growth factor (VEGF) levels, and endothelium-dependent vasodilation function (FMD) in the two groups were compared.

**Results:**

After treatment, the expression levels of NSE, TNF-α, IL-8 and CRP in the two groups were significantly decreased, and the expression levels of all indicators in the observation group were lower than that in the control group (p<0.05). After treatment, the scores of orientations, attention, memory, language, practice and action in the two groups were significantly increased, and the scores of all dimensions in the observation group were higher than those in the control group (p<0.05). After treatment, S-ICAM-1, ET-1, VEGF, FMD decreased in both groups, and all indicators of the observation group were lower than those of the control group, with statistically significant differences (p<0.05).

**Conclusion:**

Butylphthalide combined with fasudil therapy was found as effective in reducing inflammatory factors, ameliorating cognitive function and vascular endothelial function in patients with subarachnoid hemorrhage complicated with cerebral vasospasm.

## INTRODUCTION

Subarachnoid hemorrhage (SAH) is characterized by high incidence and mortality with complex mechanisms that are difficult to elucidate. Hemorrhage is mostly caused by ruptured intracranial aneurysm, accompanied by various adverse symptoms such as inflammatory reaction, cognitive impairment and impaired vascular endothelial function.^1^,^2^ Cerebral vascular spasm (CVS), as a complication of SAH, is caused by continuous constriction of intracranial arteries and decreased cerebral perfusion in the distal supply area of the supplying artery.^3^ CVS is the dominant factor leading to disability or death in patients, which can be divided into acute and delayed onset according to the characteristics of the disease course.

Prevention and treatment of CVS is the key to the prognosis of SAH. Clinically, drug therapy, hemodilution therapy and endovascular therapy are the preferred treatment methods for SAH complicated with CVS. Butylphthalide, as a new synthetic drug, has been proved to improve vascular function and can be used in the treatment of SAH complicated with CVS.^4^ Fasudil can dilate blood vessels and improve spasticity,^5^ showing an inhibitory effect on the condition of CVS. However, few studies have been carried out on butylphthalide combined with fasudil in the treatment of SAH complicated with CVS. In this study the effects of the combination of the two drugs on inflammatory factors, cognitive function and vascular endothelial function in patients were analyzed to confirm its safety.

## METHODS

In this retrospective study was used. A total of 104 patients with subarachnoid hemorrhage complicated with cerebral vasospasm admitted to Baoding First Central Hospital from July 2020 to February 2022 were selected and randomly divided into two groups by drawing lots: control group and observation group, with 52 cases in each group. In the control group, there were 24 males and 28 females, ranging from 28 to 57 years old, with an average age of (42.17±5.79) years old. Hunt-hess grading was divided into Grade-I (n=18), Grade (n=21), Grade (n=13). In the observation group, there were 27 males and 25 females, ranging from 29 to 57 years old, with an average age of (43.71±5.84) years old. Hunt-hess grading was divided into Grade-I (n=19), Grade (n=17), Grade (n=16). The study was approved by the Institutional Ethics Committee of Baoding No.1 Central Hospital (No.:2021068; date: May 27, 2021), and written informed consent was obtained from all participants.

### Inclusion criteria:

- Patients with subarachnoid hemorrhage complicated with vasospasm diagnosed by CT and MRI imaging^6^;
- Patients aged≥18, with clear consciousness, able to complete the study and signed informed consent;
- Patients with the hunt-hess score with indications for surgery^7^;
- Patients with symptoms of neurological disorders;
- Patients without allergic contraindications to butylphthalide and fasudil.

### Exclusion criteria:

- Patients with severe brain injury complicated with other etiology;
- Patients with major hematological diseases, mental disorders, and malignant tumors;
- Women in lactation or pregnancy;
- Patients with hemiplegia caused by stroke.

No statistically significant difference could be observed in the general indications (gender, age, hunt-hess grade) of the two groups (*p>*0.05), which was comparable. Patients in the two groups were treated with basic symptomatic treatment, including blood oxygen, blood pressure control, sedation, hemostasis, brain nerve disorder environment regulation, anti-epilepsy, anti-infection, lumbar puncture to drain cerebrospinal fluid, etc., while those in the observation group also received butylphthalide soft capsule (manufacturer: CSPC NBP Pharmaceutical Co., Ltd., Batch No.: State Drug Approval No. H20050299, Strength: 0.1 g) at the dosage of 0.2 g/time, tid, and fasudil hydrochloride injection (Manufacturer: Beijing Bailingwei Technology Co., Ltd., Customs Code: 29339900, Strength: 100 mg) at the dosage of 30 mg/time, tid, with two weeks as a course of treatment. During treatment, attention should be given to patients’ cranial pressure, blood pressure and infection, and targeted treatment measures should be taken.

### Observation indicators:

***Inflammatory factors:*** Eight ml of fasting venous blood was collected with a sterile coagulation tube in the early morning of the day of enrollment. After centrifugation and standing, five ml of the upper serum was taken as the sample to be tested and stored in the refrigerator at -80°C, and took out during measurement. ELISA method was used to detect the expression levels of serum neuron specific enolase (NSE), tumor necrosis factor-α (TNF-α), interleukin-8 (IL-8), and hypersensitive C-reactive protein (CRP) in the patients before and after treatment in vivo.

***Cognitive function:*** The Birmingham Cognitive Screen test (BCoS) was used for evaluation^8^, which can be divided into five dimensions: orientation (0-6 points), attention (0-54 points), memory (0-25 points), language (0-40 points), and practice and action (0-65 points). The higher the score in each dimension, the better the cognitive function of patients.

***Vascular endothelial function:*** Eight ml of fasting venous blood was collected with a sterile coagulation tube in the early morning of the day of enrollment. After centrifugation and standing, five ml of the upper serum was taken as the sample to be tested and stored in the refrigerator at -80°C, and took out during measurement. The ELISA method was used to detect serum soluble intercellular adhesion molecule-1 (s ICAM-1), vascular endothelial growth factor (VEGF) levels, and radioimmunoassay to detect blood endothelin-1 (ET-1) levels in patients before and after treatment.^9^ Endothelium-dependent vasodilation function (FMD) was measured by a high-frequency ultrasound machine.^10^ Normal values range from 7%-10%, and anything higher than 10% indicates severe blood vessel damage. All the surgeries were performed and followed by the same group of surgeons assigned for this study.

### Statistical Analysis:

All data in this study were processed by SPSS 22.0 statistical software. Measurement data (inflammatory factors, cognitive function, vascular endothelial function) were expressed as mean ± standard deviation (*χ̅*±*S*) by t test. P<0.05 indicates a statistically significant difference.

## RESULTS

Before treatment, no statistically significant differences were observed in the expression levels of NSE, TNF-α, IL-8 and CRP in the two groups (*p>*0.05). After treatment, the expression levels of NSE, TNF-α, IL-8 and CRP in the two groups were significantly decreased, and the expression levels of all indicators in the observation group were lower than those in the control group, with a statistically significant difference (*p<*0.05). Table-Ia and Ib.

**Table-Ia:** Expression levels of NSE, TNF-α, IL-8 and CRP in the two groups (*χ̅*±*S*).

Group	NSE (ug/L)		TNF-α (ng/L)	

	Before treatment	After treatment	Before treatment	After treatment
Control group (n=52)	26.17±2.56	18.17±2.04^a^	47.79±5.23	42.17±4.26^a^
Observation group (n=52)	26.23±2.61	15.23±1.15^a^	48.13±5.19	35.17±3.79^a^
t value	-0.118	9.054	-0.333	8.853
*P* value	0.906	<0.001	0.740	<0.001

***Note:***

a p<0.05 compared with that before treatment.

**Table-Ib:** Expression levels of NSE, TNF-α, IL-8 and CRP in the two groups (*χ̅*±*S*).

Group	IL-8 (ng/L)		CRP (mg/L)	

	Before treatment	After treatment	Before treatment	After treatment
Control group (n=52)	17.23±4.89	12.36±4.26^a^	56.79±7.28	46.89±5.97^a^
Observation group (n=52)	17.64±4.77	10.17±3.29^a^	57.01±7.19	40.17±4.26^a^
t value	-0.433	2.934	-0.155	6.607
*P* value	0.666	<0.001	0.877	<0.001

***Note:***

a p<0.05 compared with that before treatment.

Before treatment, no statistically significant differences were observed in BCoS scores of orientations, attention, memory, language, practice and action of the two groups (*p>*0.05). After treatment, the scores of orientations, attention, memory, language, practice and action in the two groups were significantly increased, and the scores of all dimensions in the observation group were higher than those in the control group, with a statistically significant difference (*p<*0.05). Table-IIa and IIb.

**Table-IIa:** BCoS scores of the two groups (*χ̅*±*S*, scores).

Group	Orientation		Attention		Memory	

	Before treatment	After treatment	Before treatment	After treatment	Before treatment	After treatment
Control group (n=52)	3.21±0.78	4.13±0.96^a^	31.24±6.23	36.24±5.12^a^	12.01±3.98	17.23±5.23^a^
Observation group (n=52)	3.27±0.76	5.27±0.87^a^	31.56±6.19	41.17±4.73^a^	12.04±4.01	20.17±4.97^a^
t value	-0.397	-6.348	-0.263	-5.100	-0.038	-2.939
*P* value	0.692	<0.001	0.793	<0.001	0.970	<0.001

***Note:***

a p<0.05 compared with that before treatment.

**Table-IIb:** BCoS scores of the two groups (*χ̅*±*S*, scores).

Group	Language		Practice and action	

	Before treatment	After treatment	Before treatment	After treatment
Control group (n=52)	21.17±6.23	31.89±7.23^a^	31.79±8.23	49.26±6.91^a^
Observation group (n=52)	20.99±6.30	35.74±5.79^a^	32.14±8.25	54.19±6.04^a^
t value	0.147	-2.997	-0.217	-3.874
*P* value	0.884	<0.001	0.829	<0.001

***Note:***

a p<0.05 compared with that before treatment.

Before treatment, no statistically significant differences were observed in s ICAM-1, ET-1, VEGF and FMD between the two groups (*p>*0.05). After treatment, s ICAM-1, ET-1, VEGF and FMD were all decreased in the two groups, and all indicators in the observation group were lower than those in the control group, with statistically significant differences (*p<*0.05). Table-IIIa and IIIb.

**Table-IIIa:** s ICAM-1, ET-1, VEGF, FMD of the two groups (*χ̅*±*S*).

	s ICAM-1 (ug/L)		ET-1 (pg/ml)	

	Before treatment	After treatment	Before treatment	After treatment
Control group (n=52)	419.79±62.39	368.97±42.69^a^	134.17±10.23	92.36±8.97^a^
Observation group (n=52)	417.98±65.14	321.14±39.14^a^	134.29±10.07	73.69±8.85^a^
t value	0.145	5.955	-0.06	10.684
*P* value	0.885	<0.001	0.952	<0.001

***Note:***

a p<0.05 compared with that before treatment.

**Table-IIIb:** s ICAM-1, ET-1, VEGF, FMD of the two groups (*χ̅*±*S*).

Group	VEGF (pg/ml)		FMD (%)	

	Before treatment	After treatment	Before treatment	After treatment
Control group (n=52)	215.98±23.69	169.17±12.39^a^	12.36±2.33	9.23±1.29^a^
Observation group (n=52)	215.86±22.78	117.98±11.36^a^	12.47±2.27	7.26±1.34^a^
t value	0.026	21.960	-0.244	7.633
*P* value	0.979	<0.001	0.808	<0.001

***Note:***

a p<0.05 compared with that before treatment.

## DISCUSSION

In this study, the expression of inflammatory factors such as NSE, TNF-α, IL-8 and CRP in SAH patients with CVS was significantly decreased after the combination of the two treatments, suggesting that the interaction between fasudil and butylphthalide can alleviate the symptoms of CVS and curb the occurrence of inflammation. It is documented in the literature^11^ that NSE, as a protease involved in the glycolysis process of nerve cells, is of great significance for maintaining normal physiological functions of the brain. Brain damage is often accompanied by abnormal expression of NSE, which further destroys the blood-brain barrier and easily leads to neurotoxins invading the system and inducing inflammatory reactions. TNF-α, IL-8, and CRP are all crucial response proteins involved in the immune process. Some scholars have found that^12^ TNF-α, IL-8, CRP and other inflammatory factors are overexpressed in patients with brain damage, which is consistent with the results of this study.

Blood stasis after subarachnoid hemorrhage (SAH) may give rise to vascular endothelial damage, vascular endothelin, hemoglobin, and disorder of Ca+ ion channels in blood, resulting in the occurrence of CVS.^13^ In such a case, which creates obstacles to the cognitive function of the patient’s nervous system and affects their daily life. Fasudil, as a Rho kinase inhibitor acting on CVS-induced protein kinases^14^, plays a role in suppressing the information pathway of pathogenic protein kinases. Butylphthalide, a compound extracted from parsley, has the effect of ameliorating cerebrovascular microcirculation and promoting platelet aggregation. It also inhibits excitatory amino acids from damaging the cholinergic nervous system^15^, and plays an important role in multiple stages of cerebral infarction.

Brain damage has a tendency to cause cognitive dysfunction in patients. In this study, BCoS scale test was conducted on patients in the two groups, and it was found that the observation group performed better than the control group in terms of orientation, attention, memory, language, as well as practice and action, suggesting that the interaction between fasudil and butylphthalide boasts of improving the cognitive function of patients to a large extent and benefiting the patients to resume their daily life. In case of damage to the vascular endothelium, some related proteins will be sharply expressed and released in the blood vessels.

ET-1, an active peptide synthesized by vascular endothelial cells that act on vasoconstriction, has been proved by some scholars^12^ to be over released in the process of brain damage, while promoting the influx of Ca+ ions in the blood, resulting in brain hypoxia. s ICAM-1, a protein that mediates the adhesion between vascular endothelial cells and leukocytes, is specifically involved in the pathological process of SAH. Its expression level can be employed as an important indicator for monitoring vascular endothelial damage. In case of severe damage, s ICICAM-1 increases sharply, especially in the case of acute SAH.^16^ VEGF is involved in the differentiation, growth, and migration of vascular endothelial cells, and vascular endothelial damage may lead to excessive release of VEGF.

FMD is a relatively intuitive physical indicator that reflects the level of the diastolic function of vascular endothelial cells. Studies have found that^17^ that FMD cannot be employed as an accurate indicator in the course of SAH disease due to the influence of a variety of factors, despite a certain reference value. As shown in the results of this study, the indicators of s ICAM-1, ET-1, VEGF and FMD in the observation group all decreased, suggesting that the interaction between fasudil and butylphthalide can effectively improve vascular endothelial damage, treat SAH and inhibit the occurrence of CVS.

### Limitations:

It includes fewer patients were enrolled in the study due to resource limitations. In view of this, larger sample size will be included to verify the clinical efficacy of butylphthalide combined with fasudil in patients with subarachnoid hemorrhage and cerebral vasospasm in future study

## CONCLUSION

Butylphthalide combined with fasudil has showed that it effectively reduce the level of inflammatory factors in patients with subarachnoid hemorrhage and cerebral vasospasm, ameliorate cognitive function and vascular endothelial function. Both drugs are widely applied in clinical practice due to their low price.

### Authors’ Contributions:

**NT and NG** designed this study and prepared this manuscript, are responsible and accountable for the accuracy and integrity of the work.

**CS and FS** collected and analyzed clinical data.

**JX** and **YZ**: Data analysis, significantly revised this manuscript.
